# 2432. Antibiotic Lock Therapy as a Line Salvage Strategy for Catheter-Related Bloodstream Infections: A Retrospective analysis

**DOI:** 10.1093/ofid/ofad500.2051

**Published:** 2023-11-27

**Authors:** Nischal Ranganath, Mitchell Dumais, Vaisak Nair, Hussam Tabaja, Ryan W W Stevens, John C O’Horo, Aditya Shah

**Affiliations:** Mayo Clinic, Rochester, Minnesota; Mayo Clinic Rochester, Rochester, Minnesota; Mayo Clinic, Rochester, Minnesota; Mayo Clinic, Rochester, Minnesota; Mayo Clinic, Rochester, Minnesota; Mayo Clinic, Rochester, Minnesota; Mayo Clinic, Rochester, Minnesota

## Abstract

**Background:**

Catheter-related bloodstream infection (CRBSI) is an important complication of long-term central venous catheters (CVCs) and is associated with high morbidity and cost. While line removal is optimal, it is not always feasible and is not without complications. Antibiotic lock therapy (ALT) may be an alternative strategy for line salvage, but the efficacy of this approach particularly among immunocompromised hosts is unknown.

**Methods:**

We retrospectively reviewed adult patients with CRBSI managed with ALT between 2018 and 2022 at our tertiary medical center. We reviewed patient comorbidities, type and indications for CVC use, microbiology of CRBSI, ALT, and use of systemic antibiotics. Outcomes included 30-day mortality, 90-day microbiologic relapse (with same bacteria as index case), 90-day recurrent BSI (with different bacteria from index case), and adverse events.

**Results:**

86 patients were reviewed, a majority of whom were immunocompromised (91%) due to hematopoietic stem cell transplantation (85%) and thrombocytopenic (platelet count 36; IQR 17-112). 78% had tunneled CVC for indications including peri-transplant therapy (72%), chemotherapy (15%), and TPN (6%) (Table 1). The median time to CRBSI following CVC insertion was 45 days (IQR 19-86). Infections were frequently monomicrobial (80%) due to Gram-positive organisms (n=75) including coagulase-negative staphylococci and *Enterococcus* spp. The minority of Gram-negative (n=17) infections were due to *E coli* and *Enterobacter* spp. 94% of patients received systemic antibiotics and all patients received ALT for a median of 11 days [IQR 6-15] and 11.5 days [IQR 6-14], respectively (Table 2). The 30-day attributable mortality rate was low at 1%. Rates of 90-day microbiologic relapse (20%) and recurrent BSI (16%) were high, occurring at a median of 42 days (IQR 12-55) and 35 days (IQR 25-50) following completion of ALT, respectively (Table 3).

**Figure:**
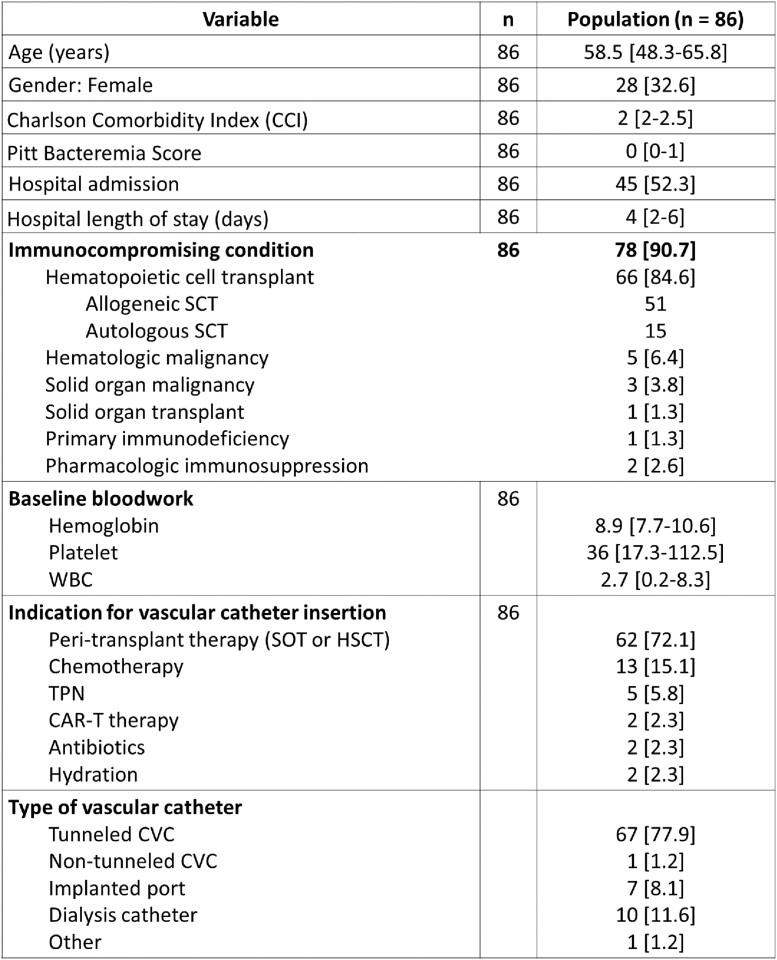

**Figure:**
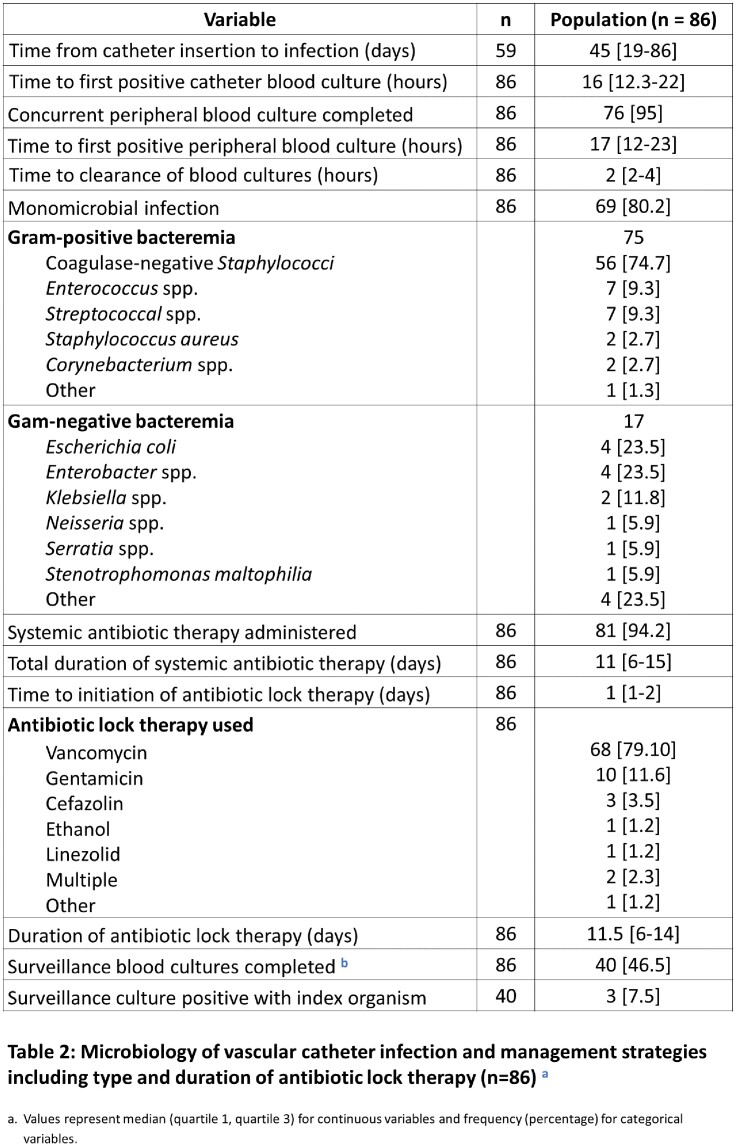

**Figure:**
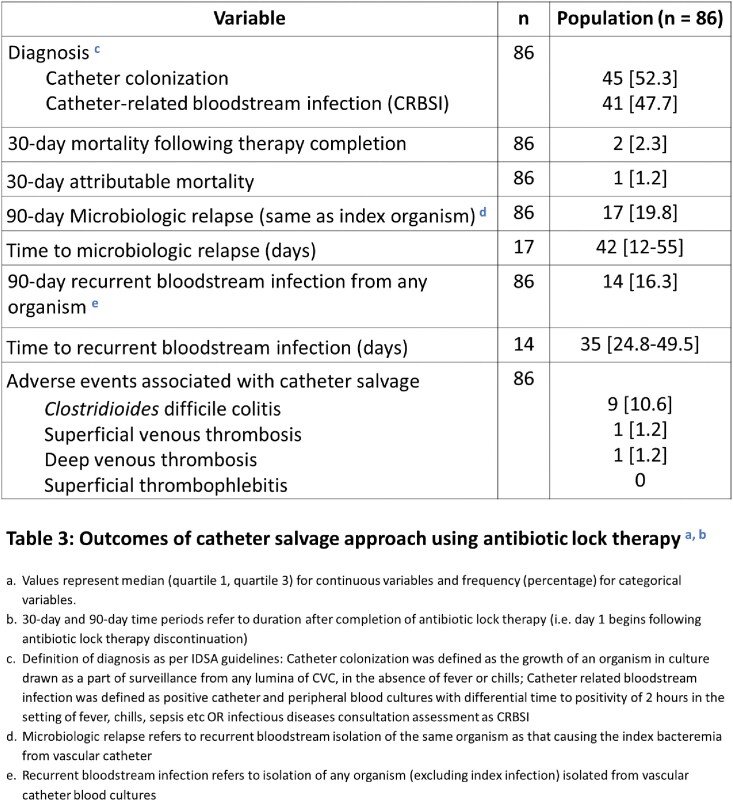

**Conclusion:**

Despite high rates of microbiologic relapse and recurrent BSI, ALT may be an effective line salvage strategy extending the use of CVC by up to 30 days with minimal impact on mortality even among immunocompromised patients.

**Disclosures:**

**John C. O'Horo, Sr., MD, MPH**, Janssen Pharmaceuticals.: Grant/Research Support|nference: Grant/Research Support

